# Addressing pregnancy‐related concerns in women with inflammatory bowel disease: Insights from the patient's perspective

**DOI:** 10.1002/jgh3.12442

**Published:** 2020-11-09

**Authors:** Emma K Flanagan, Jacqui Richmond, Alexander J Thompson, Paul V Desmond, Sally J Bell

**Affiliations:** ^1^ Department of Gastroenterology St Vincent's Hospital Melbourne Victoria Australia; ^2^ Disease Elimination The Burnet Institute Melbourne Victoria Australia; ^3^ The Australian Research Centre in Sex, Health and Society La Trobe University Melbourne Victoria Australia; ^4^ Department of Gastroenterology, Monash Health Melbourne Victoria Australia

**Keywords:** biologics, inflammatory bowel disease, pregnancy

## Abstract

**Background and Aim:**

Therapeutic options for inflammatory bowel disease (IBD) have expanded, as has the use of IBD medications in women during the reproductive period. However, no qualitative data exist that examine the pregnancy‐related concerns of women with IBD in the current era of widespread immunomodulator and biologic use. Hence, we aimed to explore in detail the impact of IBD on pregnancy from the patient's perspective.

**Methods:**

This qualitative study used semistructured interviews to explore participants' experiences regarding IBD and pregnancy until no new themes emerged. Key themes were identified using thematic analysis.

**Results:**

Fifteen women with IBD were interviewed. The majority of participants reported lingering concerns regarding their IBD medications, despite advice from their gastroenterologist that the drugs were considered safe in pregnancy. Participants more often reported medication‐related fears, such as potential negative effects on their child's immune system, than concerns regarding the effect of the disease itself on their pregnancy outcomes. A common theme was a perceived lack of knowledge among non‐IBD clinicians regarding IBD medications during pregnancy, which augmented pre‐existing anxiety.

**Conclusions:**

This study is the first of its kind to provide an in‐depth assessment of female patients' perspectives of IBD in relation to conception, pregnancy, and caring for offspring. In particular, this research characterizes the unique fears and persisting anxieties regarding IBD medications in pregnancy. The study has unearthed important insights into the specific concerns and support needs of women with IBD in order to facilitate nonjudgmental counseling designed around patient concerns and beliefs.

## Graphical abstract

This qualitative study explored the impact of inflammatory bowel disease (IBD) on pregnancy from the patient's perspective using semistructured interviews. Fifteen women with IBD were interviewed, and the majority of participants reported lingering concerns regarding their IBD medications, despite advice from their gastroenterologist that the drugs were considered safe in pregnancy. This study is the first of its kind to provide an in‐depth assessment of female patients' perspectives of IBD in relation to pregnancy and has unearthed important insights into their specific concerns and support needs in order to facilitate nonjudgmental counseling designed around patient concerns and beliefs.

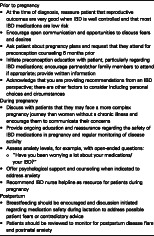

## Introduction

The prevalence of inflammatory bowel disease (IBD) is rising, and the peak incidence overlaps with child‐bearing years.^1^ Fertility is normal in patients with quiescent IBD, with the exception of women who have had pelvic surgery. The best pregnancy outcomes for women with IBD occur when their disease is in remission, for which most require maintenance medication.^2^ The therapeutic options for IBD have expanded, with increasing use of immunosuppressant and biologic medications during the reproductive period.^3^, ^4^ While the majority of IBD medications are considered safe during pregnancy and lactation,^5^ newer agents have limited pregnancy safety data, and many women harbor fears regarding harmful fetal effects.^6^

Fear of infertility and reluctance to use medication during pregnancy were prevalent in a significant proportion of Australian women with IBD in a questionnaire‐based study.^7^ However, these data were collected using physician‐designed surveys, which do not allow for dynamic patient discussion and may have missed concerns important to patients but not obvious to physicians. Another approach using telephone surveys of American women with IBD reported that over half had pregnancy concerns related to their IBD, such as the effect of IBD medications, but the nature and reasons behind these concerns were not explored.^8^

Pregnancy is a significant life event that can be associated with considerable psychological challenges, and women with IBD are particularly vulnerable to mental illness in the perinatal period.^9^ Qualitative research enables a deeper understanding of the complexity of the experiences of women with IBD and allows physicians to explore the lived experience of a health condition and gain broader insights beyond the biomedical paradigm.^10^ This is particularly crucial around the time of pregnancy, when patients have to wrestle with additional pregnancy‐related concerns and considerations, which often go unrecognized or are concealed from the treating medical team. How women with IBD feel about and experience pregnancy may influence a wide range of outcomes, including compliance with medication and, therefore, disease control; whether to have more children and when; and maternal psychological health across pregnancy. Understanding the details behind these concerns may allow IBD physicians to address these topics in their routine pregnancy counseling, acknowledge to patients that their concerns are valid, and communicate discussions with primary care and obstetric providers to educate appropriately and provide patient‐centered and consistent care for these women.

Qualitative research examining the pregnancy‐related concerns and perceptions of women with IBD in the era of widespread immunomodulator and biologic medication use does not exist.^11^ Therefore, we performed a qualitative study to explore the current impact of IBD on reproductive planning and pregnancy from the patient's perspective in order to identify key themes that should be addressed in preconception counseling and psychological care for women with IBD during the reproductive period.

## Methods

The current study used semistructured interviews to explore participants' experiences and perceptions of IBD and pregnancy. We explored the patient journey from becoming pregnant to completing their pregnancy and caring for a newborn in the setting of IBD. Participants were asked to describe their feelings about taking IBD medications during their pregnancy and while lactating and the impact of IBD on their pregnancy and peripartum experience.

Interviews were conducted between August and December 2017. Women with IBD who had been recently pregnant (within the previous 2 years) were invited to participate. Participants were recruited from the patient cohort known to the IBD service at a tertiary IBD center (St Vincent's Hospital Melbourne) through a combination of both stratified purposeful and opportunistic sampling.

Participants were given the option of completing the interview in person or via telephone. Interview questions were informed by a review of the literature and the authors' clinical experience and were developed by the multidisciplinary research team (Appendix S1). Interviews were conducted by the first author (Emma K Flanagan), who had not been involved in patient care, and were audio‐recorded and transcribed verbatim.

Interview transcripts were coded for key themes, and a coding framework was developed drawing on predetermined areas of interest and themes that emerged in the data. These emergent themes were identified using standard thematic analysis techniques; passages of text were coded by reading and reviewing the interview transcripts.^12^ This process of repeated reading and data selection was in keeping with traditional iterative coding techniques.

### *Ethical considerations*

Ethics approval was granted through St Vincent's Hospital Ethics Committee (reference number 094/17). Interviewees provided written consent to participate. Participants were not offered any reimbursement.

## Results

Fifteen participants with a confirmed diagnosis of IBD who had given birth within the last 2 years were interviewed (65% response rate). Interviews lasted approximately 45 min (range 25–65 min).

Ten women had Crohn's disease (CD), and five women had ulcerative colitis (UC). The median age at the beginning of pregnancy was 30 years (range 20–42 years), and median IBD duration was 7 years (range 4–30 years). All participants had been prescribed maintenance medication for their IBD. Eleven participants were taking a thiopurine medication (azathioprine or 6‐mercaptopurine) during pregnancy or had taken a thiopurine prior, while eight participants were prescribed biologic therapy either before or during their pregnancy.

Key themes have been reported chronologically according to the participant's pregnancy journey from preconception through to the postpartum period (Table 1). Participants' quotations are denoted by diagnosis (CD or UC) with assigned study numbers in order to preserve their anonymity.

**Table 1:** Key themes and subthemes across pregnancy

Prior to pregnancy Fertility concerns relating to IBD and IBD medications Timing of pregnancy impacted by active IBD
During pregnancy Anxiety throughout pregnancy regarding adverse pregnancy outcomes and disease flares Fears regarding effects of IBD medications on children exposed in utero Limited information from GPs and obstetric team regarding IBD
Postpartum Concerns and conflicting advice regarding breastfeeding Difficulty caring for a newborn due to IBD

GP, general practitioner; IBD, inflammatory bowel disease.

### *Prior to pregnancy*

#### *Fertility concerns relating to* *IBD* *and* *IBD* *medications*

Eight participants had concerns regarding their ability to conceive, relating both to their previous medication use and history of IBD:

We miscarried at twelve weeks… That's when we were on the [azathioprine]…For it to take seven months to fall pregnant…I didn't think that [pregnancy] was going to happen at all. Participant 14, UC

In these participants, their apprehension regarding fertility persisted at times despite being informed that their fertility would not be affected.

#### *Timing of pregnancy impacted by active* *IBD*

Five participants recognized that their IBD had impacted the timing of their pregnancy. Women reported either trying to conceive promptly during a period of disease remission due to fears of a disease flare derailing their pregnancy or having to postpone their plans for pregnancy due to active IBD:

I was always told I should…have no active illness before becoming pregnant, because of all those terrible consequences…the small, sickly baby…But… it didn't seem like there was going to be any point at which I didn't have some active disease… it was not looking like an actual reality to me, and I was getting older. Participant 13, UC

One participant also expressed her difficulties with sexual intimacy in the setting of perianal Crohn's disease:

The Crohn's affected relationship aspects as well, so everything just was put on hold…I have a recto‐vaginal fistula…[After] that big surgery…my surgeon [told] me: ‘You can't be intimate for three months’…when the time came I was really scared …I almost had the mentality where I didn't want to be intimate at all. Participant 7, CD

### *During pregnancy*

#### *Anxiety throughout pregnancy regarding adverse pregnancy outcomes and disease flares*

Seven participants expressed anxiety and persistent uneasiness relating to their IBD during pregnancy. These participants were mostly concerned regarding the possibility of a flare or complication during pregnancy or adverse outcome for their baby. As one participant explained, anxiety regarding IBD in pregnancy can be protracted:

Once I got pregnant I did become very anxious…not for myself but for my baby…I was anxious for the entire pregnancy… I kind of felt a bit cheated that I didn't enjoy my pregnancy. Participant 8, UC

#### *Concern regarding effects of* *IBD* *medications on children exposed in utero*

Twelve participants were concerned about the potentially harmful effects of their IBD medications on their babies. This was despite advice that these medications were thought to be a lower risk to the fetus than that of active IBD. Participants alluded to the universal paranoia and myriad of recommendations for pregnant women in general regarding medication use and discussed the pervasive awareness of not wanting to take any medications during pregnancy, for IBD or otherwise. Such was the influence of taking medications during pregnancy that seven participants began talking about IBD medications in pregnancy prior to being directly asked about the topic. Two women reported stopping their thiopurine preconception despite advice that is was considered safe in pregnancy.

Specific concerns regarding the use of IBD medications during pregnancy included the possibility of causing birth defects, as one participant explained:

I know there was definitely a negative connotation with the mercaptopurine, so I always thought ‘Is there residual stuff hanging around that could cause some birth defect?’… even until the day he was born I thought ‘he's going to have two heads or something’. Participant 1, CD

In addition, the possible effect on the developing baby's immune system was raised as another explanation for feeling uncomfortable with the use of IBD medications in pregnancy:

Ideally, I would like to have been on no medication…I was concerned about [my baby]… it could potentially have an impact on her in the future…some kind of an effect on suppressing her immune system… It is something that you would think of down the line if she was to develop something, could it have been as a result of taking medications while you are pregnant? Participant 9, CD

Participants commonly referred to a sense of uncertainty and not feeling reassured due to limited studies involving pregnant women exposed to IBD medications and the potential for unknown long‐term effects in the future for their children:

You're so worried about the Crohn's, and the medications, that you are instilled with a lot of fear, because the doctors don't know… studies haven't been done on women with Crohn's disease that are pregnant, so you feel like a guinea pig… I don't know the effect it's going to have on my child…If it affects me, that's fine, I'm responsible for myself, but to know that you're taking a medication that can have side‐effects, on your child…That's what worries me the most. Participant 7, CD

Participants described revised acceptance of medication during the reproductive stage due to their prevailing concern for their unborn child:

I really didn't want to be on any medication, and I know that was completely unscientific, but it was…an emotional decision, rather than an intellectual one. Being pregnant…it's like your brain's not quite so logical, you just get this sort of overwhelming, protective thing in your life, ‘I don't want low‐risk, I want no‐risk’…you would protect your baby at the expense of yourself… [the doctor]…will come up with the statistics, but…I still don't know if it's a good thing. I think they probably said that to women using thalidomide. Participant 6, CD

#### *Limited information from general practitioner and obstetric team regarding* *IBD*

Nine participants experienced a perceived lack of knowledge or conflicting advice from their general practitioner (GP) and/or obstetric team regarding their IBD and/or medications during pregnancy, which augmented pre‐existing anxiety and uncertainty. For instance, women taking a thiopurine medication were often advised it was a “Category D” or unsafe medication in pregnancy by primary care physicians or members of the obstetric team. As one participant outlined the advice she received about taking azathioprine in pregnancy (which was prescribed by her specialist and considered safe):

My normal doctor said, ‘You can't, you're on high‐risk medication’. I said, ‘No, my specialist wouldn't have prescribed it to me if I wasn't allowed on it’, and he goes ‘It's not safe, it's in the list.’ It was really stressful. Participant 14, UC

### *Postpartum*

#### *Conflicting advice regarding breastfeeding*

Nine participants expressed concern regarding breastfeeding while on IBD medications or had been given inaccurate advice from a primary care doctor or member of the obstetric team regarding the safety of their IBD medications and lactation. Some women disclosed that breastfeeding while on medications was something they felt negatively about as they felt they had more of a choice about exposing their baby to medications then than in utero.

I think breastfeeding, while taking the medication, had been more of a concern to me…I wonder if it's about him being out, and it's optional now for him to be impacted by those medications. …You can read all the studies that you want, but at the end of the day…it feels intuitive that it would have some impact, if it's passing through to him. Participant 13, UC

#### *Difficulty caring for a newborn due to* *IBD*

Nine participants raised concerns regarding a flare postpartum and their ability to care for their baby in the setting of their IBD, or had indeed faced additional challenges due to their IBD during what is already a time of immense adjustment and fatigue. Participants expressed worries about having time away from their children due to hospital admission and wanting to remain healthy for the sake of their children and described increased difficulties faced due to suffering from IBD while caring for a newborn. Four participants volunteered that they had experienced issues with bowel urgency or incontinence, particularly after pregnancy, which was then more difficult to manage when caring for young children:

Oh, it was so hard…I'd be in the middle of breastfeeding her, I'd have to put her down in her bassinet and run to the toilet… I couldn't do it on my own, like physically couldn't care for her. Participant 8, UC

## Discussion

This is the first qualitative study to evaluate the concerns facing women with IBD in relation to pregnancy in the setting of currently available IBD medications. Our study highlights that women with IBD have a range of concerns about fertility, management during pregnancy, and the postpartum period, including lactation and managing symptoms with a newborn. Their apprehension persisted despite receiving regular obstetric and tertiary IBD care.

The most pressing worry for most of these women with IBD was the potential effect of their medication, rather than their IBD itself, on their pregnancy and offspring. This was demonstrated in the majority of women expressing concern regarding IBD medication use in pregnancy and is in keeping with previous data showing that female patients report concerns about the effect of IBD medications despite good safety data for the majority of medications.^6^ Conversely, fewer women reported concerns about the adverse effect of IBD flares on the fetus, consistent with previously reported poor knowledge of the risks of spontaneous abortion, growth restriction, and premature birth.^6^

Our study details specific medication‐related fears, which included negative effects on the child's immune system, and a lack of data, meaning that women could not be “100% certain” that the medications are entirely safe for their children. These results show that, in some women, fears regarding medications persisted despite reporting adequate knowledge regarding medication safety. Participants reported a strong cultural sense that taking medications while pregnant seemed unnatural and contradicted the “maternal instinct” to “protect” their unborn children. There was a sense of distrust among participants regarding the possibility of unproven health consequences for their babies in the short term and also as‐yet‐unknown outcomes in the longer term. Moreover, these fears regarding medication existed in women with IBD regardless of disease severity or medication type. For example, women on 5‐ASA agents as monotherapy can feel apprehensive regarding medication use during pregnancy and lactation. Similarly, the majority of women in a previous Australian study assessing medication adherence in pregnancy generally believed that using any medication in pregnancy was not risk‐free.^13^

With regard to fertility, our data also show that many women with IBD are concerned about the impact of medication or disease activity when, in fact, fertility is generally normal in patients with IBD.^14^ This apprehension regarding infertility can be sustained despite being informed otherwise. The assumption that one will be infertile translates into a higher rate of unplanned pregnancies in women with chronic diseases^15^ and therefore a lack of IBD‐specific pregnancy care. Participants also worried about miscarriage once they were pregnant. Other concerns included physical symptoms related to disease flares, such as fecal urgency or incontinence and difficulties with sexual intimacy. This persisting anxiety may be partly related to women with IBD feeling “sick” and “not normal” and the public perception that this may then translate to infertility or unsuccessful pregnancies. The study uncovered a perceived incompatibility between having IBD and enduring the physicality of pregnancy and motherhood.

Compounding the anxiety among this patient group is the inconsistent information that can, at times, be provided by treating doctors. This is compounded by the fact that many of the medications used in IBD are not labeled “safe” within national medication classification systems, despite literature demonstrating safety and international society guidelines. These warnings can be raised by electronic prescribing platforms but do not take into account the clinical indication for a medication. To counteract this, the United States Food and Drug Administration is no longer using these pregnancy risk categories. However, once concerns have been raised, it is often difficult to reassure patients regarding safety.

There is a paucity of qualitative data exploring current maternal concerns among women with IBD. A recent American study that incorporated analysis of social media and online forum posts related to patients' experiences of IBD medication use during the reproductive period found themes similar to the current study.^16^ In particular, fear of IBD medications in pregnancy were highlighted, including general concerns regarding birth defects and risk of miscarriage.^16^ Likewise, this study noted that many patients seemed frustrated by insufficient knowledge from some obstetric care providers.^16^ An interview‐based study of eight Japanese women who had IBD and children ranging in age from 7 months to 7 years reported that these women predictably faced adversity at times of IBD flares.^17^ In addition, these patients also reported receiving inconsistent information from treating clinicians and an inability to find accurate information on the internet about IBD medications.^17^ One other large study that examined social media posts relating to biologic therapies in IBD found that patients expressed their concerns regarding pregnancy safety in online discussions.^18^

The novel finding of our study is the powerful knowledge generated regarding how women with IBD inherently feel about reproduction and IBD and the manner in which preconception advice is delivered, which did not always accommodate patient circumstances or preferences, even though this is essential for shared decision‐making. Our data suggest that physicians providing pregnancy information and advice for women with IBD should explore and acknowledge the patient's beliefs and use a more personal and less scientific approach, for example, with open questioning regarding specific patient concerns and how they are coping during the process of trying to conceive, throughout pregnancy, and postpartum. There was a sense that, at times, the treating clinician was not seen to recognize the importance of discussing specific maternal fears and personal circumstances during these consultations. Counseling should acknowledge our patients' potential fears, allow opportunities for them to express their concerns, provide regular reassurance, and offer referral for perinatal psychological care for anxiety when indicated (Table 2). This candid awareness of the patient experience with strong patient–physician rapport is particularly significant given the known increased risk of new‐onset mental illness in women with IBD during the perinatal period.^9^

**Table 2:** Suggested advice for patient‐centered care of women with IBD in the child‐bearing years

Prior to pregnancy At the time of diagnosis, reassure patient that reproductive outcomes are very good when IBD is well controlled and that most IBD medications are low risk Encourage open communication and opportunities to discuss fears and desires Ask patient about pregnancy plans and request that they attend for preconception counseling 6 months prior Initiate preconception education with patient, particularly regarding IBD medications; encourage partner/other family members to attend if appropriate; provide written information Acknowledge that you are providing recommendations from an IBD perspective; there are other factors to consider including personal choices and circumstances
During pregnancy Discuss with patients that they may face a more complex pregnancy journey than women without a chronic illness and encourage them to communicate their concerns Provide ongoing education and reassurance regarding the safety of IBD medications in pregnancy and regular monitoring of disease activity Assess anxiety levels, for example, with open‐ended questions: “Have you been worrying a lot about your medications/your IBD?” Offer psychological support and counseling when indicated to address anxiety Recommend IBD nurse helpline as resource for patients during pregnancy
Postpartum Breastfeeding should be encouraged and discussion initiated regarding medication safety during lactation to address possible patient fears or contradictory advice Patients should be reviewed to monitor for postpartum disease flare and postnatal anxiety

A limitation of our study is the small sample size; however, in‐depth interviews were conducted until data saturation was reached. The high response rate among a group of women with young babies to participate in an interview highlights the significance of reproductive issues for women with IBD. Although there is the potential for recall bias because the data were retrospectively collected, the study produced rich data, possibly because it provided a low‐risk context for participants to discuss their impressions, given that any findings would be anonymously reported. Women were recruited from a high‐level tertiary service providing pregnancy advice and would be expected to represent the best‐case scenario. Women with less access to counseling may have amplified concerns.

The current study has generated a thorough assessment of female patients' perspectives of IBD in relation to conception, pregnancy, and caring for offspring. In particular, this research has emphasized the unique fears and lingering anxieties regarding IBD medications in the setting of pregnancy. These important insights should be used to enhance prepregnancy counseling for women with IBD by encouraging a nonjudgmental patient‐centered approach designed around patient concerns and beliefs incorporating the themes identified in this study.

## Supporting information


**Appendix S1.** Interview schedule.
