# Supplementary material for: Addressing pregnancy‐related concerns in women with inflammatory bowel disease: Insights from the patient's perspective
Source: JGH Open. 2020 Nov 9;5(1):28–33. doi: 10.1002/jgh3.12442 (PMC7812482; doi:10.1002/jgh3.12442)
Supplement: Supplementary file 1 — Appendix S1. Interview schedule. [file JGH3-5-28-s001.pdf]

Appendix 1: Interview schedule

Background history/IBD history

1. Can you tell me about when and how you were diagnosed with IBD?

2. Can you describe what treatments you have had in the past for your IBD?

    a. Probe - tell me more about your IBD, do you know which part/s of bowel affected, any surgeries (if the participant doesn't begin to tell the story of their disease)

3. What medications are you currently taking for your IBD?

4. What physical symptoms have you had from your IBD? Do you have any of these at the moment? Do you feel like your IBD is well controlled?

5. Who lives at home with you at the moment?

    a. If children-How old are they? Do they have any current health problems? How were your pregnancies with them? (If IBD flare during pregnancy, tell me more...)

    b. If partner-How long have you been together? Do they have any health problems?

IBD and fertility/reproduction

6. Can you tell me if you are currently pregnant or planning a pregnancy? (Expand eg. how many weeks/how long have you been trying?)

7. Are/were you worried about getting pregnant? Can you tell me why that is?

8. What are your feelings about your IBD in relation to falling pregnant/being pregnant? Can you tell me why you think you feel this way?

9. How do you feel about having children? What are your main concerns?

    a. Probe – if not offered, do you worry that your child may get IBD? Do you worry about being able to care for your child during flares?

10. What impact has your IBD had on falling pregnant/being pregnant? Tell me about anything you found difficult in getting pregnant/being pregnant with IBD?

11. Probe – if not covered, has IBD affected the timing of your pregnancy? Have you had any negative comments about your chances of getting pregnant? From whom.

IBD medications

12. Have you/are you planning to make any changes to your medications during pregnancy? Can you tell me why that is? Does taking the medications worry you?

13. What are your feelings/experiences regarding breastfeeding?

14. How does/did your partner/family feel about your IBD and IBD medications when you are/were pregnant or trying to fall pregnant?

15. Have you had any negative comments about using IBD medications in pregnancy? From whom?

Information/support

16. Do you feel like you have/had enough information about IBD and pregnancy/delivery? Have you tried to find information and how successful have you been? Where did you get information from?

17. Is there any other support/information you can think of that would be helpful for women with IBD who want to get pregnant/are pregnant?

18. Are there any other thoughts you would like to share in relation to your IBD or pregnancy?
